# Design and Synthesis of Amphiphilic Graft Polyphosphazene Micelles for Docetaxel Delivery

**DOI:** 10.3390/pharmaceutics15051564

**Published:** 2023-05-22

**Authors:** Diana Serbezeanu, Tǎchițǎ Vlad-Bubulac, Ana-Maria Macsim, Vera Bǎlan

**Affiliations:** 1 “Petru Poni” Institute of Macromolecular Chemistry, 41 A Grigore Ghica Voda Alley, 700487 Iasi, Romania; 2 Faculty of Medical Bioengineering, “Grigore T. Popa” University of Medicine and Pharmacy, 9-13 Kogalniceanu Street, 700454 Iasi, Romania

**Keywords:** polyphosphazenes, histamine dihydrochloride, micelles, docetaxel, cytotoxicity

## Abstract

The structural versatility of polydichlorophosphazene derived from the inestimable possibilities to functionalize the two halogens, attached to each phosphazene main chain unit, attracted increasing attention in the last decade. This uncountable chemical derivatization is doubled by the amphiphilic roleplay demonstrated by polyphosphazenes containing twofold side-chained hydrophilic and hydrophobic moieties. Thus, it is able to encapsulate specific bioactive molecules for various targeted nanomedicine applications. A new amphiphilic graft, polyphosphazenes (PPP/PEG–NH/Hys/MAB), was synthesized via the thermal ring-opening polymerization of hexachlorocyclotriphosphazene, followed by a subsequent two-step substitution reaction of chlorine atoms with hydrophilic methoxypolyethylene glycol amine/histamine dihydrochloride adduct (PEG–NH_2_)/(Hys) and hydrophobic methyl-*p*-aminobenzoate (MAB), respectively. Fourier transform infrared spectroscopy (FTIR) and ^1^H and ^31^P-nuclear magnetic resonance spectroscopy (NMR) have been used to validate the expected architectural assembly of the copolymer. Docetaxel loaded micelles based on synthesized PPP/PEG–NH/Hys/MAB were designed by dialysis method. The micelles size was evaluated by dynamic light scattering (DLS) and transmission electron microscopy (TEM). The drug release profiles from the PPP/PEG–NH/Hys/MAB micelles were established. In vitro cytotoxicity tests of PPP/PEG–NH/Hys/MAB micelles loaded with Docetaxel revealed that designed polymeric micelles exhibited an increased cytotoxic effect on MCF-7 cells.

## 1. Introduction

Crossing the zenith point where biology and traditional polymers meet depends on an understanding of how biological systems inspire and restrain, with accuracy, the achievement of certain functions, directly from the intelligent arrangement of specific functionalities or highly ordered sequences into natural macromolecular architectures designed by nature. Nowadays, the development of such synthetic polymers with well-defined sequences and their complementary use along natural or biological polymers is impacting extensively various areas of chemistry, biochemistry, molecular biology, nanotechnology, electronics, medicine, life sciences, biomaterials etc. [1,2,3,4,5,6]. Traditionally, synthetic polymers used in healthcare applications were often borrowed from other industries without significant modifications for medical use. However, researchers in many life science fields are currently encountering significant challenges in developing materials with specific patterns of degradation profiles, biological interactions, and release characteristics, as well as physico-chemical and mechanical properties [4,7,8,9]. Many strategies to produce innovative polymers with sophisticated functions for complex activities imply the synthesis of novel monomer units with a chemical structure connected to the desired activity. However, the potential of this approach is rather limited because complicated and various functions of polymer material would require a very complex structure of monomer units, which normally result in laborous organic synthetic pathways that make the procedure more expensive and less robust. The alternate approach to be taken into consideration is to use well-known monomer units or polymers attempting to redesign a copolymer chain with given sequences of these units. The design of copolymers offers virtually limitless opportunities for variation, ranging from simple modifications of characteristics, such as monomer unit composition, average building block length, and branching availability, to more complex features, like long-range correlations or gradient structures [10,11]. Therefore, using this approach, a wide variety of novel functional copolymers could be tailored [12,13].

In the field of drug-delivery systems, polymeric micelles with a core-shell structure are receiving much attention due to their unique drug loading form, as well as their disposition characteristics in the body [14,15,16]. Polymeric micelles have a size in the nanoscale range, rendering them highly suitable as drug-delivery carriers that evade renal exclusion and the reticuloendothelial system. Additionally, their small size imparts an improved vascular permeability. The polymer base plays a crucial role in the development of polymeric micelles for drug-delivery applications [17,18,19]. The selection of polymers for drug delivery should ideally prioritize biocompatibility and the ease of customization or functionalization. However, there is a growing need for these polymers to meet increasingly complex sets of properties and design parameters in line with advancements in clinical science. The development of next-generation biomedical polymers must incorporate a range of desirable features, such as the interaction with specific biological targets, environmental responsiveness, modulated degradation, and the formation of supramolecular assemblies. Yet most synthetic macromolecules used in the biomedical area have not been designed originally for these specific applications, and they lack the desired chemical flexibility. The use of phosphorus-based materials, such as polyphosphates, polyphosphonates, and polyphosphazenes, has gained significant interest in the biomedical field due to their biodegradability, blood compatibility, reduced protein adsorption, and strong interactions with dentin, enamel, or bones. In particular, polyphosphazenes (PPP), which are macromolecules that feature a phosphorus and nitrogen backbone, are well-suited to achieve these objectives [20]. When compared to more widely used biomaterials like polycaprolactone and poly(lactic acid), polyphosphazenes have a distinct advantage for drug delivery because the halogen atoms can easily be replaced by other molecules or macromolecules via post-polymerization functionalization. As a result, multifunctionalized main-chain polyphosphazenes can be designed with a variety of physical, chemical, and biological properties. The induced excellent conformational flexibility, deriving directly from the variety of hydrophobic and hydrophilic substituents, allows the nanoaggregation of hydrophobic segments and governs the formation of amphiphilic nanosized micellar or polymersomes-like structures. The hydrophilic groups tend to orient themselves towards the aqueous environment, where they can interact with water molecules, while the hydrophobic groups seek to clump together and reduce their contact with water when the polymer is placed into an aqueous environment. As a result, self-assembled nanostructures like micelles or vesicles are created, where the hydrophilic groups form a shell on the surface and interact with the water, and the hydrophobic groups are confined to the core of the nanostructure, away from the surrounding water. Several variables, including the size and shape of the hydrophobic groups, the length of the hydrophilic chains, the overall molecular weight, and the architecture of the self-assembled nanostructures, affect the size and stability of the nanostructures. Such well-defined macomolecules demonstrated superior stability in comparison to liposomes, and, as a consequence, they have drawn attention as vesicles for drug-delivery application [21,22,23,24].

It is noteworthy to mention that for nanomedicine use purposes (for example, tissue engineering and targeted drug delivery), the side groups attached to the PPP backbone should be hydrolytically sensitive and biocompatible. Thus, PEGylation of PPP has been adopted, lending several desirable properties such as biocompatibility, plasma-solubility, the capability of protecting the drug inside the vesicle, and preventing protein adsorption while “travelling” to the targeted area (stealth effect) [25]. PEG (polyethylene glycol) can improve the plasma-solubility of polymers by increasing their hydrophilicity. PEG is a highly water-soluble and biocompatible polymer that can be conjugated to other hydrophobic polymers to create a hydrophilic surface that is more easily soluble in water-based environments like blood plasma. Additionally, PEGylation can improve the circulation time of polymers in the bloodstream by reducing their recognition by the immune system and avoiding rapid clearance by the liver and kidneys. This is because PEGylation can create a steric hindrance around the polymer, preventing it from being recognized by opsonins and other immune cells that would typically target it for removal from the bloodstream. The increased circulation time can improve the effectiveness of drug-delivery systems by allowing more time for the drug to be released from the polymer and reach its target site.

Besides its non-toxicity and nature-inspired building-block capability, histamine has drawn attention due to its stimuli responsive character. Consequently, it has been observed that uncharged imidazole groups generate hydrophilicity in basic or neutral fluids like blood, but charged imidazole groups induce hydrophobicity in environments with an acidic pH like endosomes or tumor locations [26], thus promoting the endosomal escape of the vesicle carrying the chemotherapeutics and increasing the efficiency of the treatment. Docetaxel, a semisynthetic agent also inspired by nature, derived from *Taxus baccata* L. genus. It demonstrated its anti-cancer efficiency in the treatment of solid tumors but still faced the disadvantage of its scarce water solubility [27,28].

The main objectives of the current work were to design and synthesize a new amphiphilic graft polyphosphazene (PPP/PEG–NH/Hys/MAB) to investigate the capability of the composition with adjusted balance between hydrophilicity and hydrophobicity to encapsulate Docetaxel in micelle-like structures, and to study its drug loading and release behavior. The chemical structure of the synthesized PPP/PEG–NH/Hys/MAB was characterized by nuclear magnetic resonance (NMR) and FTIR. The self-assembly and micellization behavior of PPP/PEG–NH/Hys/MAB were evaluated by dynamic light scattering (DLS) and transmission electron microscopy (TEM). The encapsulation of Docetaxel into polymeric micelles derived from functionalized polyphosphazenes was performed. Then, in-vitro release and cytotoxicity studies on the resulted drug loaded micelles were interpreted and discussed.

## 2. Materials and Methods

### 2.1. Materials

Hexachlorocyclotriphosphazene (99.95%) was purchased from Sigma-Aldrich (Steinheim, Germany) and purified by sublimation at 80–90 °C before use. Aluminum chloride (AlCl_3_, Reagent Plus, 99%), methoxypolyethylene glycol amine (PEG–NH_2_, molecular weight 2000, extent of labeling: ≥ 0.4 mmol/g NH_2_ loading), histamine dihydrochloride (Hys, ≥99.0% (AT)), methyl-*p*-aminobenzoate (MAB, 98%) and Docetaxel (purum, ≥97.0% (HPLC)) were acquired from Sigma-Aldrich (Steinheim, Germany) and used without further purification. All other reagents were purchased from commercially available sources and used without further purification. Adriamycin-resistant MCF-7 human breast cancer cell line was acquired from the European Collection of Cell Cultures (ECACC).

### 2.2. Synthesis of PPP/PEG–NH/Hys/MAB

**Scheme 1:**
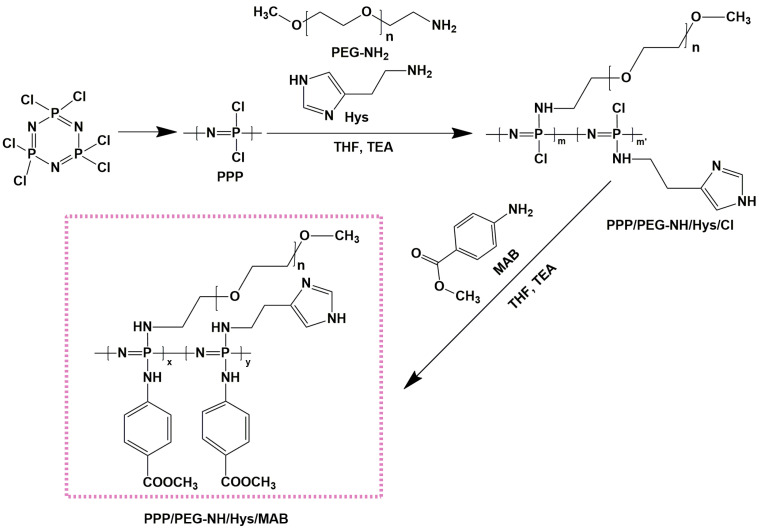
Synthesis of PPP/PEG–NH/Hys/MAB.

Poly(dichlorophosphazene) was produced through the thermal ring opening polymerization of hexachlorocyclotriphosphazene in the presence of 3 wt.% anhydrous AlCl_3_ at 250 °C for a period of 4–5 h (Scheme 1) [29].

The polymer was purified by dissolving the mixture in toluene and precipitation from dry petroleum ether [30,31]. Dry THF solution (3.35 mL) containing PEG–NH_2_ (0.5 g, 0.25 mmol), histamine (0.26 g, 2.34 mmol), and dry triethylamine (0.24 mL, 1.72 mmol) was added dropwise into 0.5 g poly(dichlorophosphazene) and dissolved in 2.7 mL of THF, while the mixture was magnetically stirred at room temperature for 12 h. Then, an excess amount of MAB (1.28 g, 8.46 mmol), dissolved in 11.52 mL of THF in the presence of triethylamine (1.18 mL, 8.45 mmol), was added slowly to complete the substitution reaction of the second chlorine atom and the mixture was continuously stirred for additional 24 h, filtered off, and subjected to solvents removal in a rotary evaporator. The resulting product was poured into diethyl ether to obtain a precipitate. Afterwards, the obtained polymer was dialyzed (MWCO: 10.500) in distilled water for 48 h and freeze-dried.

### 2.3. Preparation of Drug-Loaded Polymeric Micelles

**Scheme 2:**
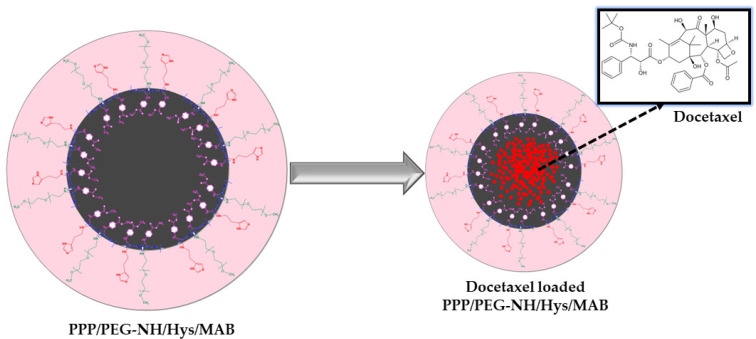
Graphical representation of Docetaxel loaded into PPP/PEG–NH/Hys/MAB micelles.

Depending on the medication kind, and the particular micelle system being employed, the procedure for loading pharmaceuticals into micelles can vary. Typically, the medication and the micelle-forming agent(s) are both dissolved in a suitable solvent. The subsequent stirring or sonication of the mixture causes the production of micelles, which trap the drug inside the hydrophobic core of the micelle. In our study, Docetaxel was loaded into PPP/PEG–NH/Hys/MAB micelles, using the dialysis combined with ultrasonic methods [32,33]. 5 mL solution of Docetaxel at 0.1% in DMSO were dropped into 50 mL of PPP/PEG–NH/Hys/MAB solution under ultrasonication for 5 min (Bandelin Sonopulus HD 2200, Berlin, Germany, output power 50 W, temperature −5 °C). Afterwards, the Docetaxel-loaded PPP/PEG–NH/Hys/MAB micelles were dialyzed against deionized water, ultrasonicated for another 2 min, and then freeze-dried. The same procedure was used for the preparation of drug-free PPP/PEG–NH/Hys/MAB micelles (Scheme 2).

### 2.4. Characterization

#### 2.4.1. NMR Studies

^1^H and ^31^P NMR spectra were recorded on a Bruker Avance 400-MHz spectrometer (Bruker, Rheinstetten, Germany) at room temperature using D_2_O as solvent.

#### 2.4.2. FTIR Spectroscopy

FTIR spectroscopy was used to analyze both the copolymer and drug-loaded micelles. The KBr pellet technique was employed for this purpose. The spectra were obtained using a Bruker VERTEX 70 device (Santa Barbara, CA, USA) and ranged from 4000 to 500 cm^−1^, with a resolution of 4 cm^−1^.

#### 2.4.3. Dynamic Light Scattering (DLS)

The particles size and size distribution of the PPP/PEG–NH/Hys/MAB micelles were determined by DLS (Malvern ZetasizerNanoS (Malvern Instruments, Worcestershire, UK).

#### 2.4.4. Transmission Electron Microscopy (TEM)

To examine the morphology of the micelles and drug-loaded micelles, a Hitachi HT7700 transmission electron microscope (Hitachi, Tokyo, Japan) operating at 100 kV was utilized. The samples were prepared by ultrasonication in water, and a single drop of the resulting dilute dispersions was deposited onto a carbon-coated grid. The solvent was then evaporated in an oven at 40 °C for 72 h. Subsequently, the samples were analyzed using high-resolution mode. The diameter statistics of the micelles and drug-loaded micelles were determined from five TEM images using ImageJ program (Version 1.53t 24 August 2022 (upgrade) [34].

#### 2.4.5. Docetaxel Loading Content of PPP/PEG–NH/Hys/MAB Micelles

The Docetaxel loaded inside the PPP/PEG–NH/Hys/MAB micelles was determined by the UV-VIS spectroscopy. For this purpose, 4 mg of freeze-dried PPP/PEG–NH/Hys/MAB were dissolved in 10 mL of DMSO, then the UV-VIS intensity at 229 nm was measured (UV-1700 PharmaSpec, Shimadzu, Manassas, VA, USA) and the amount of free Docetaxel (W_free_) was determined using a previously established standard curve. The Docetaxel loading (DL (%)) and the Docetaxel loading efficiency (EE (%)) were calculated using Equations (1) and (2).

DL (%) = ((W_added_ − W_free_)/W_Docetaxel loaded into PPP/PEG–NH/Hys/MAB micelles_) × 100 (1)

EE (%) = ((W_added_ − W_free_)/W_added_) × 100 (2)

where W_added_ and W_free_ represent the amount of Docetaxel initially added and the amount of free Docetaxel in the supernatant.

#### 2.4.6. In-Vitro Release of Docetaxel Loaded PPP/PEG–NH/Hys/MAB

Docetaxel release from PPP/PEG–NH/Hys/MAB micelles were carried out using the dialysis method [32] in two phosphate-buffered saline (PBS) media (7.4 and 5.5). Typically, 1 mL of micellar solution was seeded in a dialysis bag (Spectrum MWCO 12,400 Da, Sigma-Aldrich, Munchen, Germany) that have been placed in preheated PBS (20 mL) and incubated at 37 °C. Periodically, samples were taken and subjected to UV-VIS analysis (n = 3), and fresh buffered solutions were added.

#### 2.4.7. Cytotoxicity Evaluation of PPP/PEG–NH/Hys/MAB Micelles and Docetaxel Loaded PPP/PEG–NH/Hys/MAB

MCF-7 cells were grown in DMEM medium with 10% fetal bovine serum and 1% antibiotics. A number of 5 × 10^3^ cells/well were cultivated in 96-well plates at 37 °C, a humidified atmosphere, 5% CO_2_. Docetaxel-loaded micelles and blank micelles, suspended in the DMEM medium at different concentrations, were added. A MTT assay was performed at 24 h and 48 h.

## 3. Results and Discussion

The main objectives of creating this copolymer were to enhance the stability of micelles and enable a controlled release of docetaxel. It consists of a hydrophilic PEG–NH_2_ chain and a hydrophobic Hys/MAB chain. Under acidic conditions, the Hys/MAB chain can switch to a hydrophilic state, facilitating the release of the encapsulated cargo. Thus, the novel copolymer PPP/PEG–NH/Hys/MAB was prepared by grafting hydrophilic and hydrophobic side groups on poly(dichlorophosphazene), as shown in Scheme 1. Poly(dichlorophosphazene) dissolved in THF and reacted with methoxypolyethylene glycol amine (PEG–NH_2_) and histamine (Hys) to yield copolymer (PPP/PEG–NH/Hys/Cl). In addition, an excess amount of methyl-*p*-aminobenzoate (MAB) reacted to yield copolymer PPP/PEG–NH/Hys/MAB [29].

**Figure 1:**
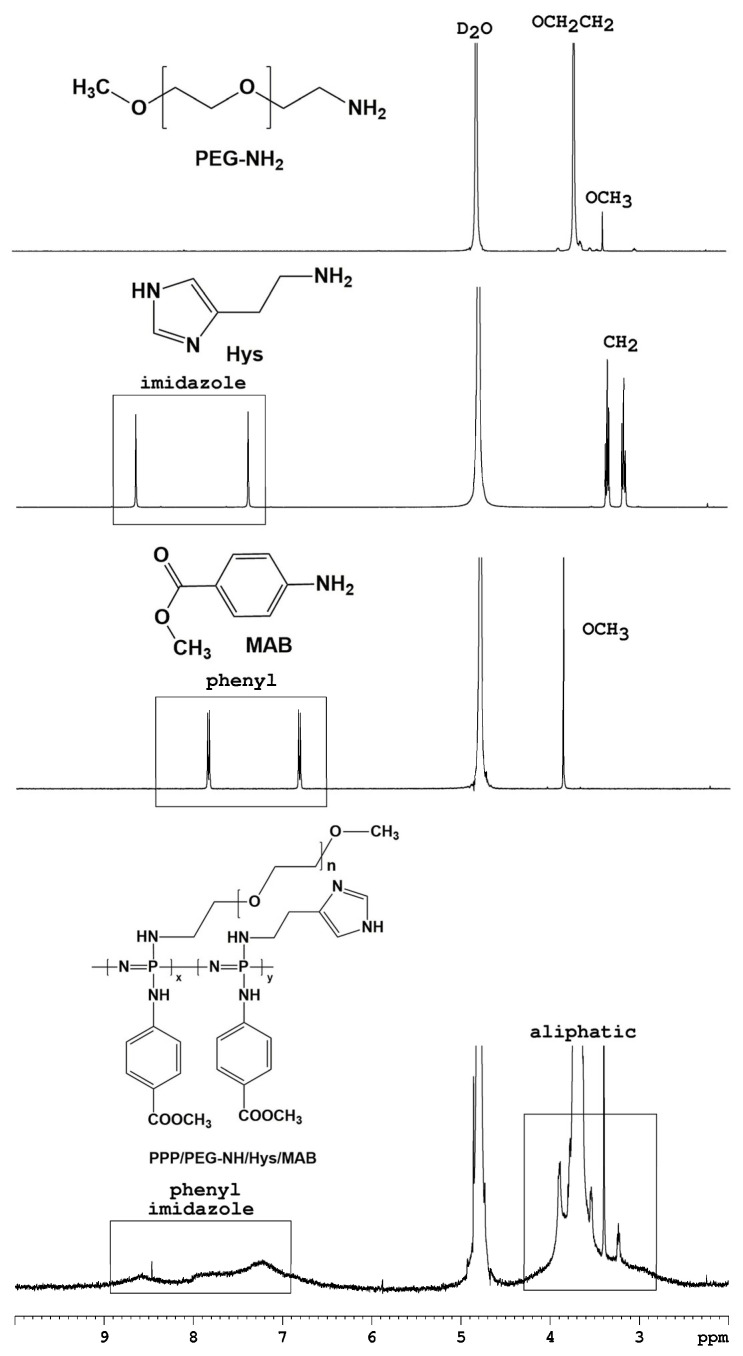
^1^H NMR spectra of PEG–NH_2_, Hys, MAB, and PPP/PEG–NH/Hys/MAB.

**Figure 2:**
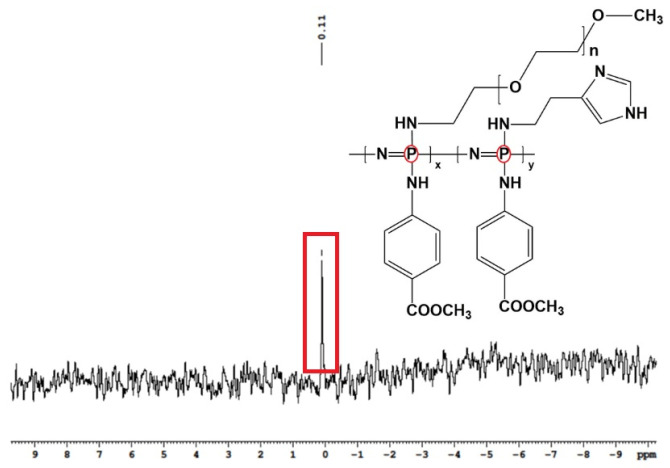
^31^P NMR spectrum of PPP/PEG–NH/Hys/MAB.

**Figure 3:**
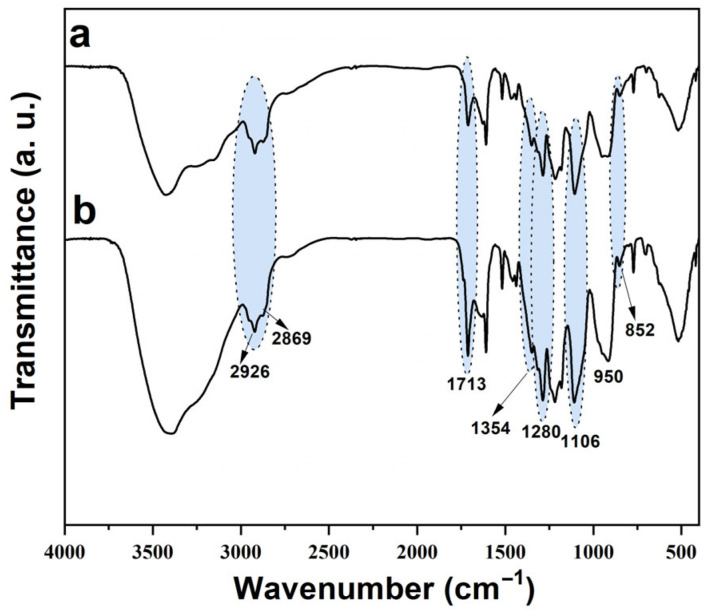
FTIR spectra of PPP/PEG–NH/Hys/MAB (**a**) and Docetaxel-loaded PPP/PEG–NH/Hys/MAB micelles (**b**).

The formation of the copolymer PPP/PEG–NH/Hys/MAB was confirmed by NMR and FTIR spectroscopy (Figure 1, Figure 2 and Figure 3). The ^1^H NMR spectra of PEG–NH_2_, Hys, and MAB, compared with the spectrum of PPP/PEG–NH/Hys/MAB, are shown in Figure 1. In addition to PEG–NH_2_ signals, the new peaks related to both the Hys and MAB (with little difference on the ratio of the integral for the signals) were observed. So, the ^1^H NMR spectrum of the PPP/PEG–NH/Hys/MAB showed peaks at 6.6–8.6 ppm and 3–4.1 ppm characteristics to the aromatic and aliphatic protons, respectively. In Figure 1, it can be observed that the peaks of aromatic protons in MAB and Hys groups appeared in the range of 6.6–8.6 ppm, while those at 3.7 ppm (–O–CH_2_–CH_2_–) and 3.3 ppm (–OCH_3_) corresponded to the protons of the PEG group [23]. The molecular composition of the copolymer PPP/PEG–NH/Hys/MAB was determined based on the ratios of the integral of the methoxy protons in PEG (s, 3.3) to the integral of the signal of the –CH_2_ protons in Hys (s, 3.23) to the integral of the –CH_3_ protons of MAB (d, 3.8) (Figure 1). It was found that the composition ratio of PPP/PEG–NH/Hys/MAB resembled the feed ratio.

In the ^31^P NMR spectrum of copolymer, the peak at 0.11 ppm is ascribed to the phosphorus resonances of the –N–P–N– unit (Figure 2, red box). Taking into account all this data (Figure 1 and Figure 2), it can be concluded that a new amphiphilic graft polyphosphazene, decoded as PPP/PEG–NH/Hys/MAB, was synthesized.

The FTIR spectrum of PPP/PEG–NH/Hys/MAB (Figure 3a) exhibited the typical absorption peaks at 2926 and 2869 cm^−1^ (–CH_2_–, asymmetric and symmetric stretching vibrations), 1713 cm^−1^ (C=O, stretching vibration), 1600 and 1524 cm^−1^ (aromatic C–C, stretching vibration), 1465 cm^−1^ (–CH_2_–, deformation vibration), 1280 cm^−1^ (C–O, stretching vibration), 1106 cm^−1^ (C–O–C, stretching vibration), and 852 cm^−1^ (*p*-substituted benzene vibration). The P=N stretching bands of backbone were observed at 1354 cm^−1^, and the absorption bands characteristic to the P=N stretching vibration were noted at 950 cm^−1^ (Figure 3a).

As presented previously in the literature [35], the FTIR spectrum of Docetaxel showed absorption bands of O–H at 3480 cm^−1^, C=O stretching of ester group at 1741 cm^−1^, and a band corresponding to C–O at 1109 cm^−1^. In Figure 3b, the FTIR spectrum of the Docetaxel-loaded miceles reveals a series of characteristic bands at 3369 cm^−1^ for ν_N-H_ and ν_OH_, at 1710 cm^−1^ for ν_C=O_ and at 1596 cm^−1^ for δ_N-H_) [36]. It was assumed that Docetaxel was incorporated inside of the hydrophobic core of the amphiphilic PPP/PEG–NH/Hys/MAB micelles. Therefore, its bands could be overlapped with those of polymer. (This is information that was also published by other authors [37]).

### 3.1. Particle-Size Analysis

**Figure 4:**
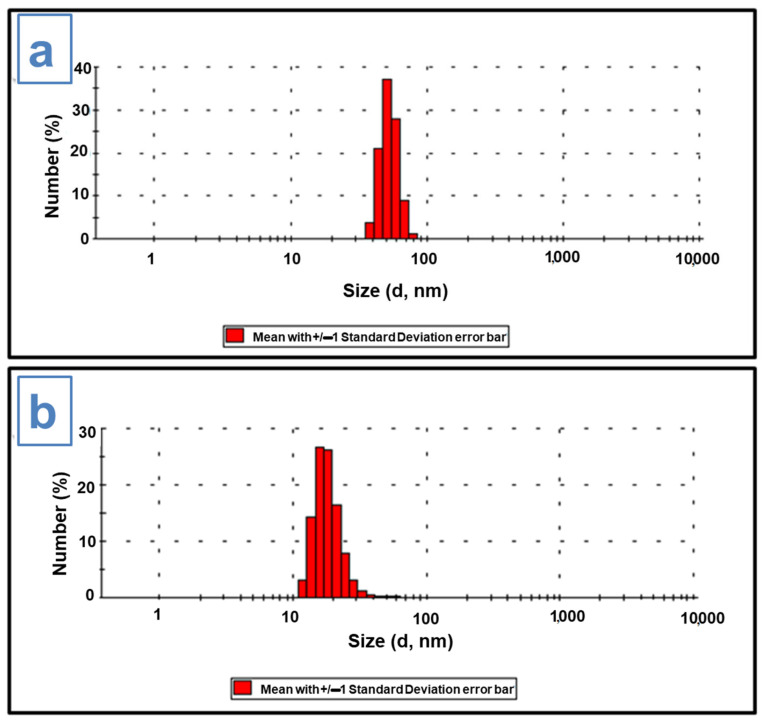
Size distribution of empty polymeric micelles (**a**) and size distribution of Docetaxel-loaded micelles (**b**).

Using the DLS (dynamic light diffusion) technique, the average hydrodynamic size of the drug-loaded micelles, compared to the empty micelles, was measured. The DLS results showed a monomodal distribution and an average diameter of 40–50 nm for the empty micelles (Figure 4a), as well as a diameter of 20–30 nm for the drug-loaded micelles (Figure 4b), indicating that the encapsulation of the Docetaxel decreased the micelles size, compacting the nanostructure through its hydrophobic interactions with the inside polymeric core. According to studies, when Docetaxel is introduced to a solution that forms micelles, it can interact with the hydrophobic core of the micelle, making it more compact and smaller [38]. This is due to the fact that docetaxel has a sizable hydrophobic domain that might take up space in the micelle’s core, reducing the micelle’s overall size. Docetaxel can also interact with the micelle’s surface, making it more stable and lowering the likelihood that it will combine and create larger structures. Overall, the presence of Docetaxel may result in a reduction in the size of micelles and an improvement in their stability, which may be advantageous for uses like drug delivery. These results were also sustained by TEM micrographs.

The zeta potential value for the Docetaxel-loaded micelles was −8.66 mV, while for the drug-free micelles, it registered a value of −10.5 mV.

### 3.2. Transmission Electron Microscopy Study

**Figure 5:**
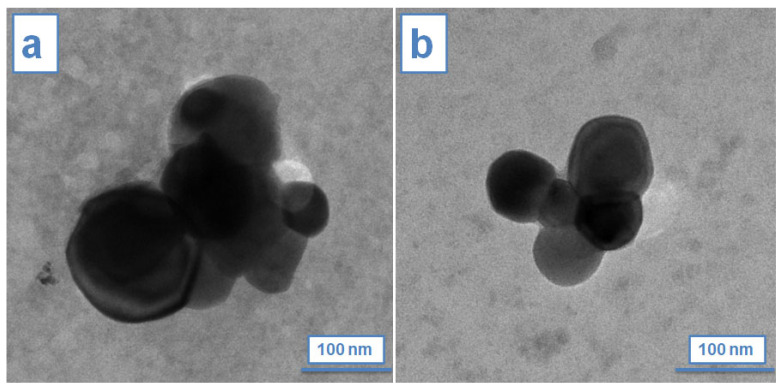
TEM images corresponding for empty polymeric micelles (**a**) compared with Docetaxel-loaded micelles (**b**).

TEM was used to study the micelles morphology (Figure 5a,b). As it can be observed in Figure 5a, the empty micelles adopted clustered irregular spherical shapes, with an average diameter of approximately 97 ± 41 nm (Figure 5a), while the Docetaxel-loaded micelles measured an average diameter of 70 ± 16 nm (Figure 5b). The incorporation of the hydrophobic drug Docetaxel into the hydrophobic micelle core may have caused a reduction in the amount of space within the core, which may explain why polymeric micelles shrank in size after drug loading. As a result, the polymer chains in the core region rearrange themselves in a more compact manner to accommodate the drug molecules, causing the overall size of the micelles to shrink. Additionally, the presence of the drug in the core of the micelles may induce changes in the structure or organization of the polymer chains, resulting in a more tightly packed and condensed micelle structure. This behavior was also described in other recent scientific papers, where the authors explained that the decreasing of drug-loaded micelles size is a consequence of the hydrophobic micellar interactions with drug molecules [39].

The differences observed in the sizes of the micelles measured by two techniques (DLS and TEM) could be attributed to the distinctive features of the experimental methods used. Specifically, DLS measurements were performed on aqueous suspensions, while TEM analyzed the dry form of the micelles. Furthermore, due to the influence of absorption forces, the polymer self-assembly may have collapsed on the TEM grid. Due to their size (with an average diameter of 70 ± 16 nm), Docetaxel-loaded PPP/PEG–NH/Hys/MAB micelles exhibit great potential as drug release systems for targeted cancer therapy.

### 3.3. Drug Release Studies

**Figure 6:**
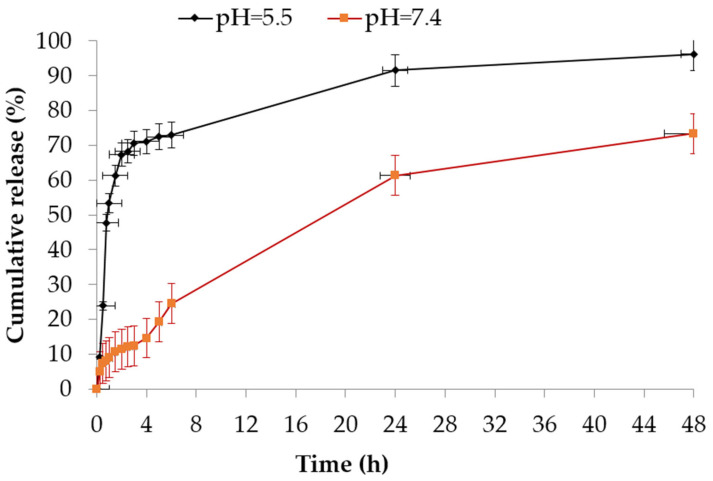
Release profile of Docetaxel from PPP/PEG–NH/Hys/MAB micelles. Studies performed in vitro in pH = 5.5 and pH = 7.4.

A drug-loading efficiency of 82.7% was determined from Equation (2), a value that indicates a high efficiency, as well as the fact that Docetaxel was successfully incorporated into the structure. To assess the suitability of PPP/PEG–NH/Hys/MAB micelles as controlled drug-release systems, for the evaluation of PPP/PEG–NH/Hys/MAB micelles as potential controlled drug release systems, the release tests have been conducted in two media (a buffer with pH = 7.4) that mimic the normal cells, and also a buffer with pH = 5.5 for cancer cell environment simulation (Figure 6). The outcomes of these experiments, presented in Figure 6, evidenced a faster release of the Docetaxel from the polymeric micelles in the first 4 h (a cumulative release of 70% in pH = 5.5 compared with 15% in pH = 7.4), pursued by a maintained release for the next 48 h. The rapid release of the drug can be attributed to the diffusion of the drug located on the surface of the micelles or near its hydrophilic part.

From Figure 6, it can also be observed that the release of the drug loaded in the micelles was much faster in pH = 5.5 compared to pH = 7.4, with this data indicating that these micelles could be used primarily for tumor cells that have an environment that is more acidic than that of normal cells. In addition, the faster release from the acidic environment can be governed by the reprotonation of amino groups in the structure of the drug, including the charging of the imidazole ring from hystamine moieties, which may be responsible for activating the degradation of the interior of the micelles that can occur at lower pH values [26]. Consequently, the polymer becomes more positively charged, resulting in a greater electrostatic repulsion among particles and leading to their expansion. As the pH of blood is around 7.4, the micelles should remain stable in blood. Therefore, pH-sensitive micelles will only release their contents within cells, reducing the risk of off-target toxicity and increasing drug accumulation in the tumor site. Based on these findings, it can be concluded that pH-sensitive micelles are a promising strategy for targeted drug delivery.

### 3.4. MTT Assays

The biocompatibility of materials refers to the reaction of materials in contact with the tissues of the human body in accordance with their medical applications and represents the ability of a material to render a response close to the tissue to which it adheres [40]. The first step was to establish if empty micelles exert any cytotoxic effects upon the MCF-7 mammary cell line. Cytotoxicity refers to the ability of a substance to cause damage or death to cells. MCF-7 is a breast cancer cell line widely used in research, so it is often employed as a model system for testing the cytotoxic effects of various substances on breast cancer cells. There are many factors that can influence the cytotoxic effects of a substance on MCF-7 cells, including the concentration of the substance, the duration of exposure, and the specific properties of the substance itself. Known chemotherapy drugs like doxorubicin and paclitaxel also showed cytotoxic effects on MCF-7 cells by interfering with the division and replication of cancer cells.

**Figure 7:**
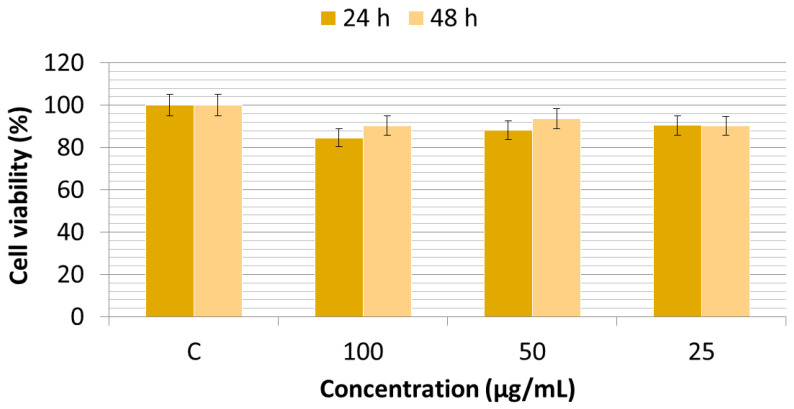
MTT test for the PPP/PEG–NH/Hys/MAB micelles.

The results of the MTT test, (Figure 7) showed that after 48 h of incubation, the proportion of metabolically active (live) cells is greater than 80%, which indicates that the empty micelles are not cytotoxic in the concentration range tested.

**Figure 8:**
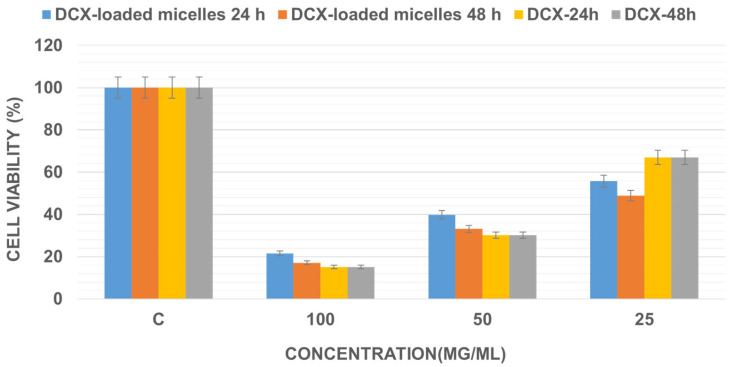
MTT test for Docetaxel drug and Docetaxel loaded PPP/PEG–NH/Hys/MAB micelles.

In the following stage, the viability of the breast cell line MCF-7 was tested in contact with the PPP/PEG–NH/Hys/MAB micelles loaded with Docetaxel, compared with the drug alone. The results of the MTT test, presented in Figure 8, revealed that drug-loaded micelles led to a drastic decrease of cell viability after 24 h. For the concentration of 25 µg/mL, a gradual decrease in cell viability is observed, starting from 55% after 24 h, while for the highest tested concentration (100 µg/mL), a cell viability of 22% was recorded after 24 h, (the viability decreases to 17% after 48 h). Similarly, Docetaxel exhibits a meaningful cytotoxic impact. MTT data are in accordance with drug-release profiles; the substantial decrease of the cellular viability observed at 24 h is corelated with the same period, in which the major quantity of the Docetaxel was released.

The study demonstrated that docetaxel-loaded micelles were more cytotoxic compared to free docetaxel at a concentration of 25 mg/mL. This enhanced efficacy could be attributed to the amphiphilic nature of the micelle, which functions similar to surfactants, thus promoting the mobility of the micelles and their diffusion into tumor cells. Once taken up by cells, the micelles dissociate in the acidic lysosomal environment, facilitating intracellular accumulation of docetaxel. Furthermore, the polymer micelles may inhibit the expression of P-glycoprotein (P-gp) pump, thus preventing drug efflux and increasing the intracellular drug concentration, leading to improved antitumor activity [41,42,43,44]. Further understanding of these effects requires detailed investigations of the self-assembly processes and release mechanisms in complex media containing proteins.

## 4. Conclusions

The study aimed to develop passive targeting micelles for cancer therapy by investigating a new type of amphiphilic polyphosphazene that contains methoxy polyethylene glycol, histamine, and MAB. The formation of self-assembled systems of amphiphilic micelles was highlighted by TEM. The potential of the micelles to be used as vehicles for antitumor drugs was subsequently tested. The investigation included examining the self-aggregation behavior of copolymers in water, encapsulating drugs, studying the in-vitro release of the drug, and assessing the cytotoxicity of anti-cancer agent-loaded micelles. The MTT test findings revealed that after 48 h, the percentage of metabolically active cells (i.e., viable cells) was above 80% for empty micelles at the tested concentration range, indicating their lack of toxicity, whereas micelles loaded with Docetaxel exhibited a high cytotoxic effect. The study also showed that these micelles were capable of significantly inhibiting the proliferation of MCF-7 tumor cells in vitro, demonstrating a more potent disruption of cell growth compared to free docetaxel. Overall, the cytotoxic effects of PPP/PEG–NH/Hys/MAB micelles loaded with Docetaxel on MCF-7 cells provided valuable insights into its potential use as an anti-cancer agent, but careful consideration must be given further to the specific conditions of the experiment and the properties of the substance being considered for specific applications.

## Data Availability

The data that support the findings of this study are contained within the article.
